# Circulating GFAP and Iba-1 levels are associated with pathophysiological sequelae in the thalamus in a pig model of mild TBI

**DOI:** 10.1038/s41598-020-70266-w

**Published:** 2020-08-07

**Authors:** Audrey D. Lafrenaye, Stefania Mondello, Kevin K. Wang, Zhihui Yang, John T. Povlishock, Karen Gorse, Susan Walker, Ronald L. Hayes, Patrick M. Kochanek

**Affiliations:** 1 Department of Anatomy and Neurobiology, Virginia Commonwealth University Medical Center, P.O. Box 980709, Richmond, VA 23298 USA; 2 Department of Biomedical and Dental Sciences and Morphofunctional Imaging, University of Messina, Via Consolare Valeria, 98125 Messina, Italy; 3 Oasi Research Institute-IRCCS, Troina, Italy; 4 Program for Neurotrauma, Neuroproteomics and Biomarkers Research (NNBR), Department of Emergency Medicine, Psychiatry and Neuroscience, McKnight Brain Institute, University of Florida, 1149 Newell Drive, Gainesville, FL 32611 USA; 5 Banyan Laboratories, 13400 Progress Boulevard, Alachua, FL 32615 USA; 6 Department of Critical Care Medicine, Safar Center for Resuscitation Research, University of Pittsburgh School of Medicine and Children’s Hospital of Pittsburgh of UPMC, 4401 Penn Avenue, Pittsburgh, PA 15224 USA

**Keywords:** Astrocyte, Microglia, Diseases of the nervous system, Fluorescence imaging, Animal disease models, Biochemical assays

## Abstract

Serum biomarkers are promising tools for evaluating patients following traumatic brain injury (TBI). However, their relationship with diffuse histopathology remains unclear. Additionally, translatability is a focus of neurotrauma research, however, studies using translational animal models are limited. Here, we evaluated associations between circulating biomarkers and acute thalamic histopathology in a translational micro pig model of mTBI. Serum samples were collected pre-injury, and 1 min-6 h following mTBI. Markers of neuronal injury (Ubiquitin Carboxy-terminal Hydrolase L1 [UCH-L1]), microglial/macrophage activation (Ionized calcium binding adaptor molecule-1 [Iba-1]) and interleukin-6 [IL-6]) and astrogliosis/astrocyte damage (glial fibrillary acidic protein [GFAP]) were measured. Axonal injury and histological features of neurons and glia were also investigated using immunofluorescent labeling and correlated to serum levels of the associated biomarkers. Consistent with prior experimental and human studies, GFAP, was highest at 6 h post-injury, while no substantial changes were observed in UCH-L1, Iba-1 or IL-6 over 6 h. This study also found promising associations between thalamic glial histological signatures and ensuing release of Iba-1 and GFAP into the circulation. Our findings suggest that in diffuse injury, monitoring serum Iba-1 and GFAP levels can provide clinically relevant insight into the underlying acute pathophysiology and biomarker release kinetics following mTBI, providing previously underappreciated diagnostic capability.

## Introduction

Traumatic brain injury (TBI) is an increasing challenge and a global health priority^1^, with more than 50 million TBIs occurring world-wide each year with an associated cost of approximately $400 billion^2^. The vast majority (~ 95%) of patients suffer a mild TBI (mTBI) with Glasgow Come Scale (GCS) scores of 13–15^3,4^ which, contrary to common perception, cause structural sequelae with variable degrees of injury to neurons, glia, and vascular structures, leading to a spectrum of potential clinical outcomes. As translatability is a primary focus of neurotrauma research, there has been a call for use of higher-order gyrencephalic animal models prior to transitioning to the clinic^5–9^, however, knowledge regarding the TBI-induced pathophysiology in higher order animal models is still limited. Operation Brain Trauma Therapy (OBTT) is a drug- and biomarker-screening consortium intended to address barriers in the translation from preclinical to clinical studies in TBI by improving the quality of preclinical studies and providing a rigorous framework to increase the translational potential of experimental TBI treatments. The approach taken by OBTT incorporates heterogeneous types of brain injuries, sensitive histological and biomarker outcome measures and rigorous standardized protocols to ensure reliability and reproducibility across multiple institutions^10,11^. In addition, the workflow of the consortium dictates that the most effective therapies and biomarkers found in OBTT’s rodent studies move to testing in a higher order gyrencephalic model of TBI. Due to their high level of homology with humans in terms of systemic inflammatory responses, metabolic rates, and cytoarchitecture, we utilized an adult micro pig model to study the effects of mild TBI in a more translational fashion^12–15^.

Serum biomarkers are promising tools to evaluate patients after TBI noninvasively and many studies have demonstrated correlations between biomarker levels and clinical outcome or gross pathological findings to inform prognostication of patients with severe TBI^16–18^. Within the unique OBTT multi-center, multi-species, pre-clinical therapy and serum biomarker screening consortium framework^8,10^, we have demonstrated that certain biomarkers, namely glial fibrillary acidic protein [GFAP], can be considered as a surrogate endpoint of gross pathology and strongly predicts behavioral morbidity and response to therapies across multiple experimental rodent models of TBI^8,11^. Clinical studies have also found serum biomarker levels of GFAP and Ubiquitin Carboxy-terminal Hydrolase L1 (UCH-L1) to strongly predict outcome and to correlate to gross brain pathology in the human population^8,17,19,20^. Additionally, clinical studies have found associations between magnetic resonance imaging (MRI) signatures indicative of diffuse pathology and levels of glial fibrillary acidic protein GFAP and/or UCH-L1^21–24^. Additionally, biomarkers focusing on inflammation, such as Ionized calcium binding adaptor molecule 1 (Iba-1), and interleukin 6 (IL-6) are just beginning to be explored. However, little is known about how variations in mTBI-induced diffuse pathobiological manifestations correlate to alterations of circulating biomarker levels, sparking debate regarding the trajectory and scope of the utility of biomarker assessments for mTBI and concussion^25^.

Diffuse axonal injury (DAI) in white matter has historically been considered the primary pathological hallmark of mTBI, though, growing clinical evidence supports the idea that thalamic damage plays a central role in the pathogenesis of various symptoms of mTBI^26–28^. Nonetheless, potentially owing to the challenges in visualizing thalamic damage following mTBI, few studies have investigated thalamic injury. Therefore, the pathoanatomical features and pathophysiological mechanisms underlying thalamic injury following mTBI remain to be defined. Previous research from OBTT found DAI within various brain regions following central fluid percussion injury (cFPI) in micro pigs at 6 h following mTBI with significant and consistent invovlement of the thalamic domain^29^. We also detected acute microglia/macrophage activation in the thalamus following mild TBI in micro pigs, suggesting a role for inflammation following mTBI in this model^29,30^. These pathophysiological changes were observed in the absence of cell death or parenchymal micro bleeds within the thalamic domain^29^, indicating that this micro pig model is ideal for assessing potential diffuse mTBI-induced thalamic patholphysiology.

In the current study, we aimed to expand the previous OBTT findings^29^ to examine the potential associations between specific aspects of mTBI-induced diffuse thalamic damage and circulating serum GFAP and UCH-L1 biomarker levels. Hence, serum biomarker levels were assessed using our micropig model of cFPI and were compared to histological alterations previously found within the thalamic domain^29^. As mTBI is usually associated with little to no contusional load, but does demonstrate DAI^31,32^, we assessed a marker of neuronal damage, UCH-L1, which has been shown to be upregulated in serum acutely following TBI clinically and that might be useful in distinguishing between focal and diffuse injuries^17,20,33,34^. Additionally, we investigated whether histological evidence of thalamic glial injury/alteration is associated with serum levels of a panel of circulating biomarkers following mTBI, by interrogating a variety of pathophysiological mechanisms in both serum and thalamic tissue, namely microglia/macrophage activation, using Ionized calcium binding adaptor molecule 1 (Iba-1), and astrocytic injury, using GFAP. Given its role in neuro-inflammation after TBI, we also assessed serum levels of IL-6. This study represents a step toward our ultimate goal, which is to determine whether or not serum biomarkers might represent useful tools for future investigation to inform on the impact of therapies on the response to injury.

## Results

### Temporal profile and characterization of circulating biomarkers in the micro pig following cFPI

**Figure 1:**
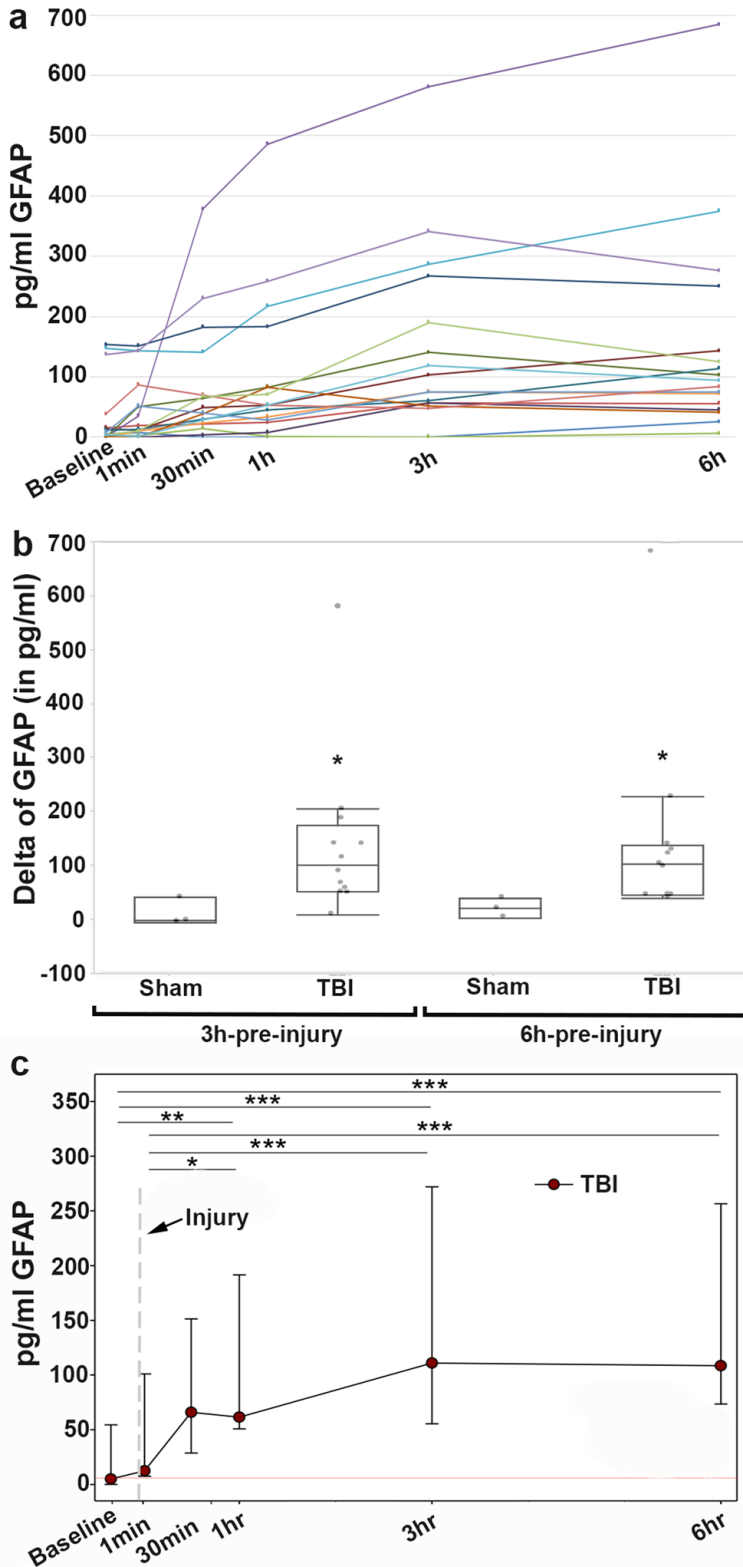
Serum concentrations of the astroglial biomarker, glial fibrillary acidic protein (GFAP), increased over the first 6 h following cFPI. (**a**) Serum GFAP levels of individual animals throughout the pre and 6 h post-injury time points with each colored line representing an individual sham or cFPI animal. (**b**) Box plots of the change in serum GFAP level from pre-injury to 3 hr post-injury (“3 hr-pre-injury”) or change in serum GFAP level from pre-injury to 6 hr post-injury (“6 h-pre-injury”) in sham-injured (n = 3) or cFPI (n = 14) micropigs. Each grey circle represents an individual animal. Note that while there was a significant increase in serum GFAP levels over both 3 h and 6 h following cFPI, sham levels remained rather unchanged. (**c**) Line graph depicting the medians ± interquartile range concentration of GFAP in the serum of pigs at pre and post-injury time points. Sham levels were consistent throughout the time course and were excluded from the graph. The time of cFPI is indicated by the dashed line. While serum GFAP levels were consistent among pre-injury baseline, and acutely post-cFPI, GFAP levels at 6 h following cFPI were significantly increased compared to baseline and 1 min post-cFPI. *p < 0.05, **p < 0.01, ***p < 0.001.

The serum biomarker results were compared both to baseline values as well as longitudinally over the 6 h post-injury time frame. Circulating GFAP levels consistently increased over time in all pigs sustaining cFPI, demonstrating a significant increase in the change of GFAP levels from pre-injury (ΔGFAP) at both 3 h (Mann–Whitney U Test, sham n = 3, cFPI n = 14; p = 0.006) and 6 h (p = 0.003) compared to sham (Fig. 1). In the injured population, GFAP levels rose over time and were highest at 6 h after injury (median 98.65 pg/mL), demonstrating an approximately eightfold increase compared to levels at 1 min post-injury (median 12.5 pg/ml, *p* < 0.05) and pre-injury baseline levels (median 11.5 pg/ml, *p* < 0.01) (Fig. 1c). This high level of serum GFAP at 6 h after injury is consistent with prior experimental and human work demonstrating a peak of GFAP levels acutely (hours) following injury^20,35–37^.

**Figure 2:**
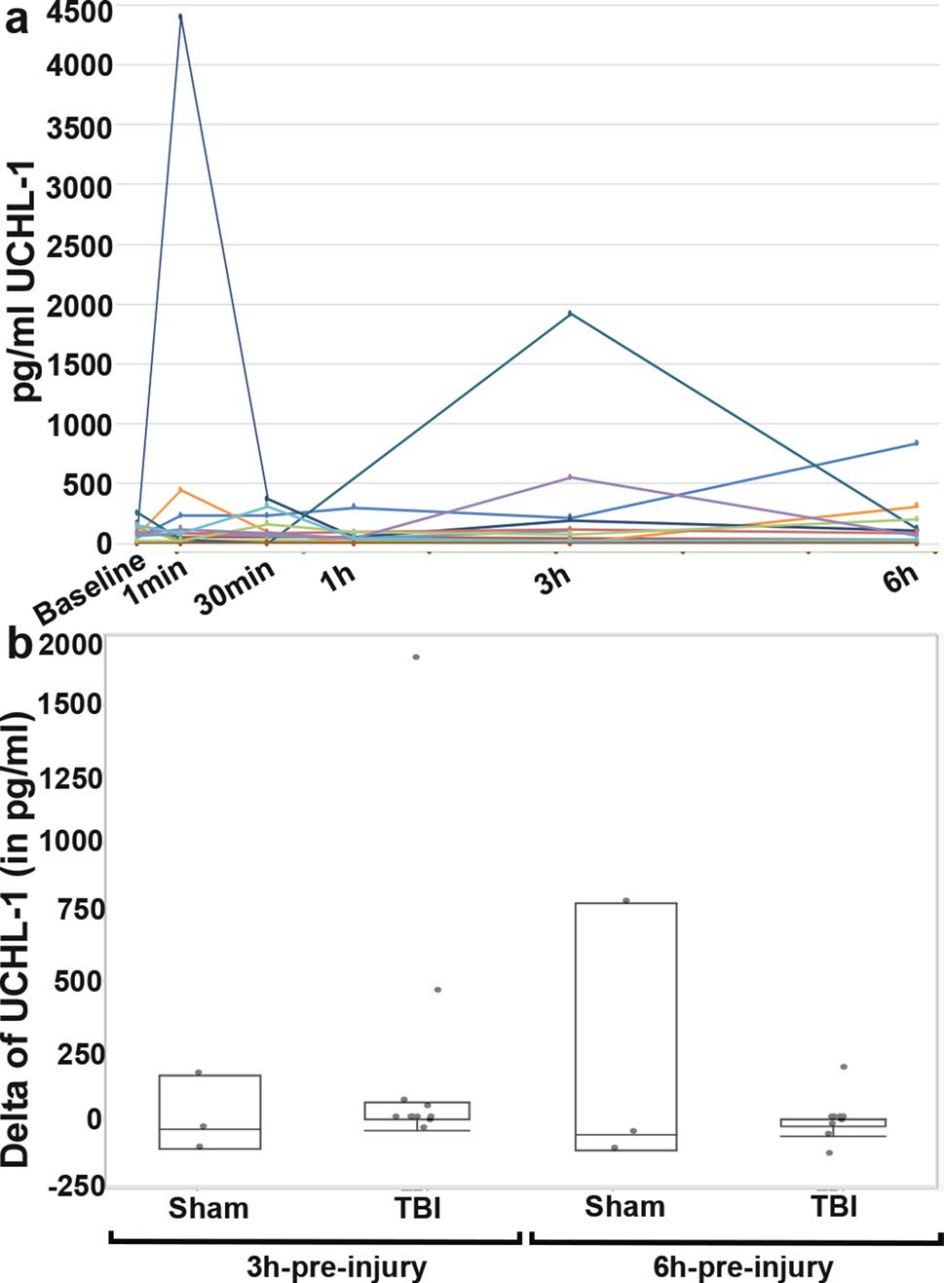
Serum levels of UCH-L1 following central fluid percussion or sham injury. (**a**) Line graph depicting the concentration of UCH-L1 in the serum of individual pigs at pre-injury and 1 min, 30 min, 3 h and 6 h post-injury. Each colored line represents an individual sham or cFPI animal. (**b**) Box plots of the average change in serum UCH-L1 level from pre-injury to 3 hr post-injury (“3 hr-pre-injury”) or change in serum UCH-L1 level from pre-injury to 6 hr post-injury (“6 hr-pre-injury”) in sham-injured (n = 3) or cFPI (n = 14) micropigs. Each grey circle represents an individual animal. Note that there were no significant differences in UCH-L1 serum levels or delta between sham and cFPI micro pigs.

**Figure 3:**
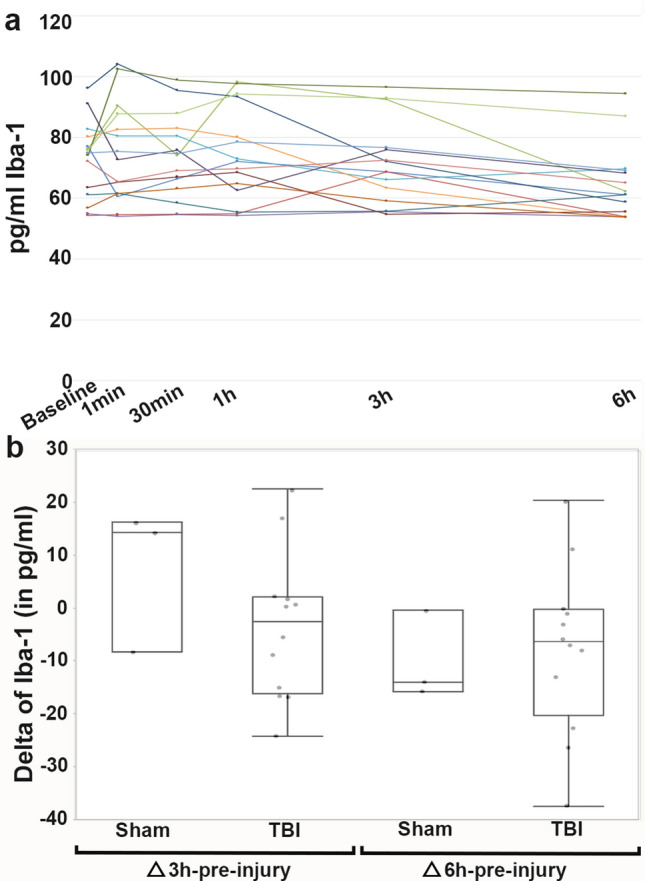
Serum levels of Iba-1 prior to and 1 min, 30 min, 3 h and 6 h following cFPI or sham injury. (**a**) Line graph depicting the concentration of Iba-1 in the serum of individual animals with each colored line representing an individual sham or cFPI animal. (**b**) Box and Whisker plots of the average change in serum Iba-1 level from pre-injury to 3 hr post-injury (“3 hr-pre-injury”) or change in serum Iba-1 level from pre-injury to 6 hr post-injury (“6 hr-pre-injury”) in sham-injured (n = 3) or cFPI (n = 12) micropigs. Each grey circle represents an individual animal. Note that there were no differences in Iba-1 serum levels between sham and cFPI micro pigs.

**Figure 4:**
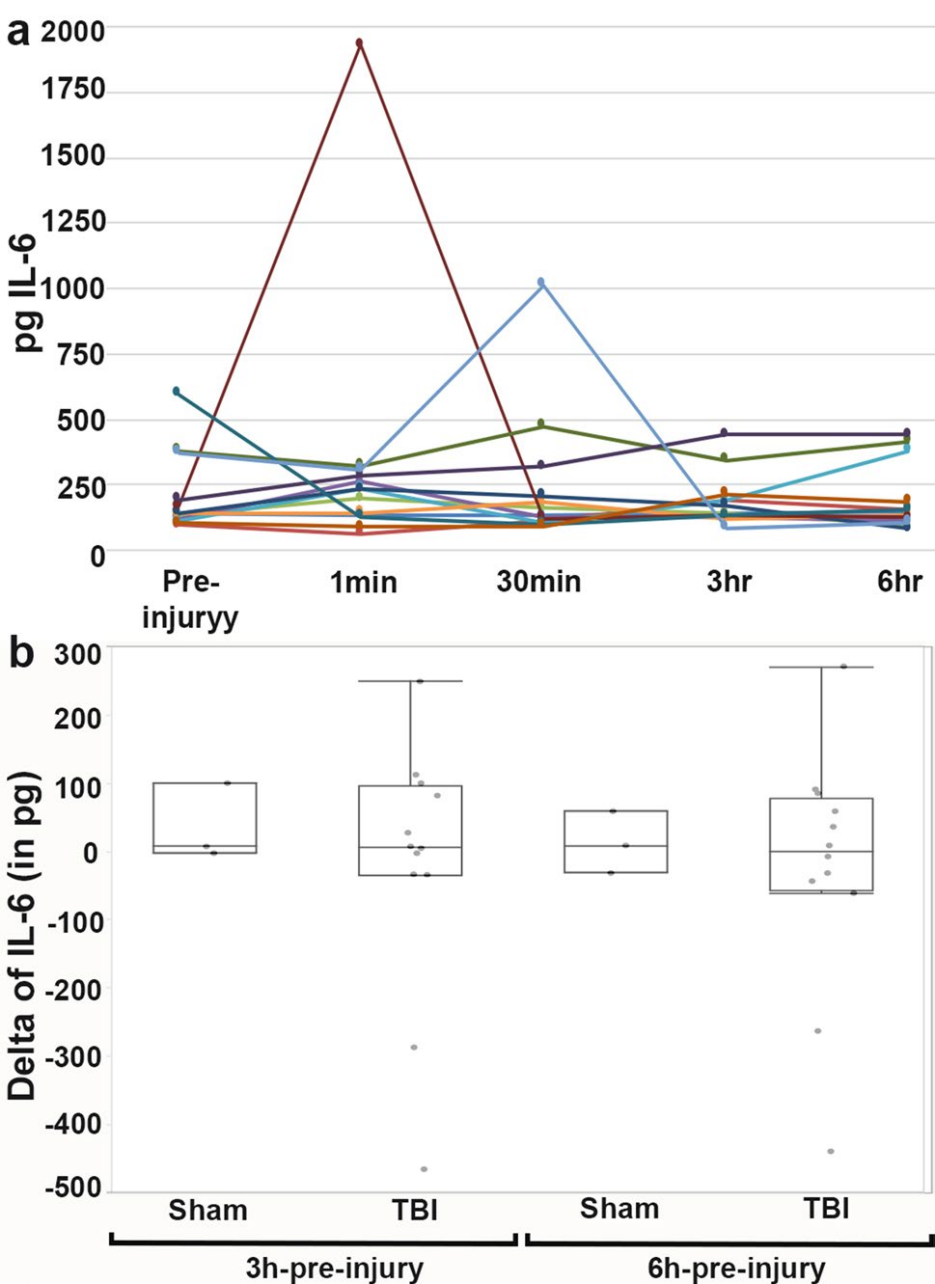
Serum levels of IL-6 prior to and 1 min, 30 min, 3 h and 6 h following cFPI or sham injury. (**a**) Line graph depicting the concentration of IL-6 in the serum of individual animals with each colored line representing an individual sham or cFPI animal. (**b**) Box and Whisker plots of the average change in serum IL-6 level from pre-injury to 3 hr post-injury (“3 hr-pre-injury”) or change in serum IL-6 level from pre-injury to 6 hr post-injury (“6 hr-pre-injury”) in sham-injured (n = 3) or cFPI (n = 10) micropigs. Each grey circle represents an individual animal. Note that there were no differences in IL-6 serum levels between sham and cFPI micro pigs.

Unlike GFAP, there was no evidence of longitudinal changes in UCH-L1 (Fig. 2), Iba-1 (Fig. 3) or IL-6 (Fig. 4) serum levels over the first 6 h following cFPI and no substantial change in the levels of UCH-L1, Iba-1 or IL-6, in post-injury samples compared with baseline samples.

### Blood brain barrier permeability in the thalamus is altered by cFPI in the pig

**Figure 5:**
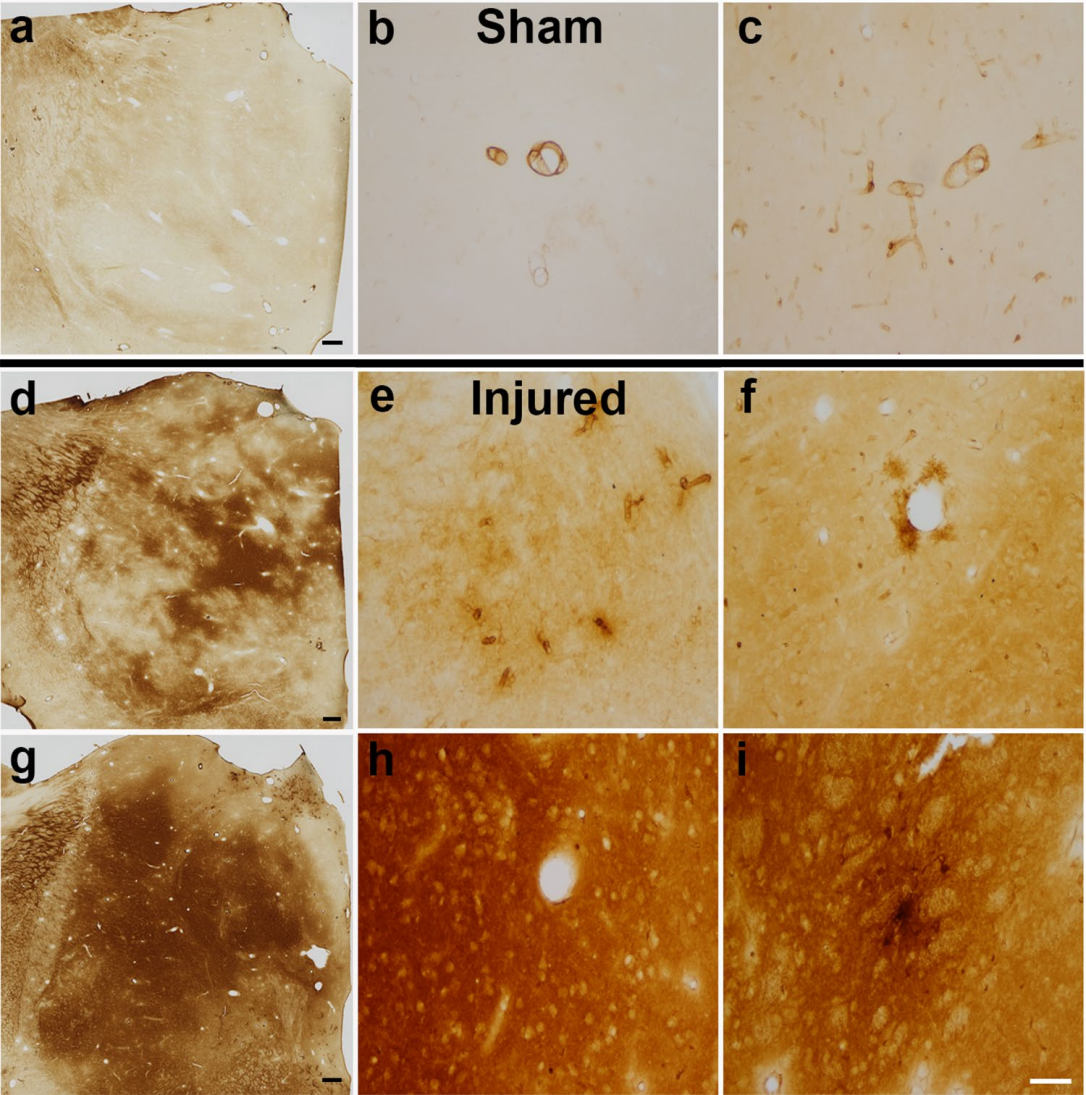
The blood brain barrier is compromised 6 h following cFPI in the thalamus of micro pigs. Light photomicrographs labeled for swine serum albumin of thalami from (**a**–**c**) sham or (**d**–**i**) cFPI micro pigs 6 h post-injury. In sham-injured tissue serum albumin is restricted to the blood vessels. Following cFPI, however, it is diffusely distributed within the thalamic tissue following TBI. (**d**–**f**) In some areas of the injured thalamus serum albumin was observed in a patchy distribution and/or within the parenchyma surrounding blood vessels. Other areas of the injured thalamus or (**g**–**i)** the entire thalamus of some injured animals demonstrated serum albumin labeling throughout the thalamic parenchyma. (**f**,**i**) Cells containing serum albumin with glial morphology were also detectable following cFPI. Scale **a**,**d**,**g**: 500 μm; **b**,**c**,**e**,**f**,**h**,**i**: 100 μm.

Thalamic blood brain barrier (BBB) disruption following injury has recently been demonstrated in various experimental TBI models^38–40^. To investigate the occurrence of BBB disruption in our micro pig model of cFPI, we assessed the localization of serum albumin within the thalamus 6 h following sham or injury. While serum albumin was exclusively found within the vasculature in sham-injured micro pigs, abnormal albumin accumulation was observed in the thalamic parenchyma of all injured animals (Fig. 5). Parenchymal albumin accumulation, however, varied among animals in terms of both localization and intensity. In some injured thalami albumin was primarily observed in direct apposition to blood vessels (Fig. 5c), while others demonstrated serum albumin labeling throughout the thalamic parenchyma (Fig. 5d). Similar to observations made by Johnson et al.^40^, we also detected cells with glial morphology that had taken up serum albumin (Fig. 5e,f).

### Circulating levels of UCH-L1 do not correlate with thalamic axonal pathology or UCH-L1 histological features in cFPI

**Figure 6:**
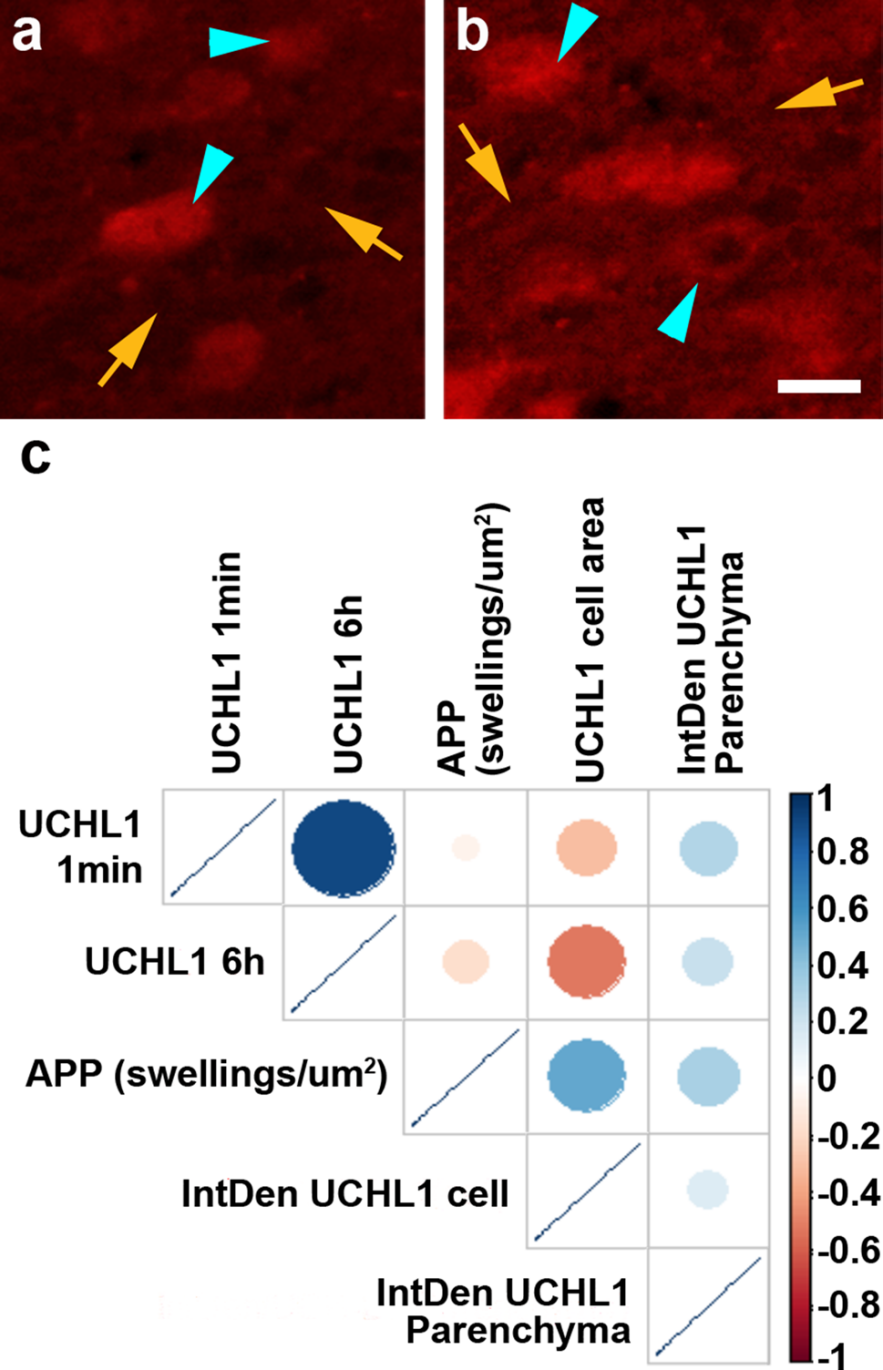
Circulating levels of UCH-L1 with histochemical correlations of thalamic neuronal injury 6 h following cFPI. (**a**,**b**) Representative images demonstrating the UCH-L1 + neuronal cells (blue arrow heads) and the parenchyma (yellow arrows) assessed. **(c**) Correlation matrix (generated in R^78^) depicting the Spearman Rho correlation for each pair of assessment values on the y and x axis with blue indicating a stronger positive correlation and red indicating a stronger negative correlation and white being no correlation. There were no discernable correlations between UCH-L1 serum biomarker levels and histological features following injury. Scale: 20 μm.

To investigate potential injury-effects on neuronal pathology we previously assessed both DAI and neuronal injury in the thalamic domain. We found that cFPI in micro pigs produced significant DAI within the thalamic domain 6 h post-injury without apparent cell damage/death^29^. Here we furthered this investigation, by assessing potential alterations in UCH-L1 labeling 6 h following cFPI. We did not find any differences in UCH-L1 + neuronal number (One-way-ANOVA with Bonferroni posthoc; sham n = 2, cFPI n = 11 animals; F_1,11_ = 0.004, p- = 0.95), neuronal size (F_1,11_ = 0.034, p- = 0.86) or UCH-L1 localization (F_1,11_ = 0.342, p- = 0.57) between sham and injury. To determine if UCH-L1 serum levels might be associated with axonal injury, we investigated whether the degree of DAI within the thalamus was associated with serum levels of UCH-L1, but no correlation was found (Fig. 6). We further explored the relation of UCH-L1 serum concentrations with histological features of UCH-L1 + neurons, including the expression levels of UCH-L1 in individual neurons or the surrounding parenchyma (Fig. 6). Again, no correlation was found between serum UCH-L1 levels at either 3 h or 6 h following injury and any of these histological outcomes.

### Morphological features of acute microglia/macrophage activation within the thalamus are associated with serum levels of Iba-1 but not IL-6

**Figure 7:**
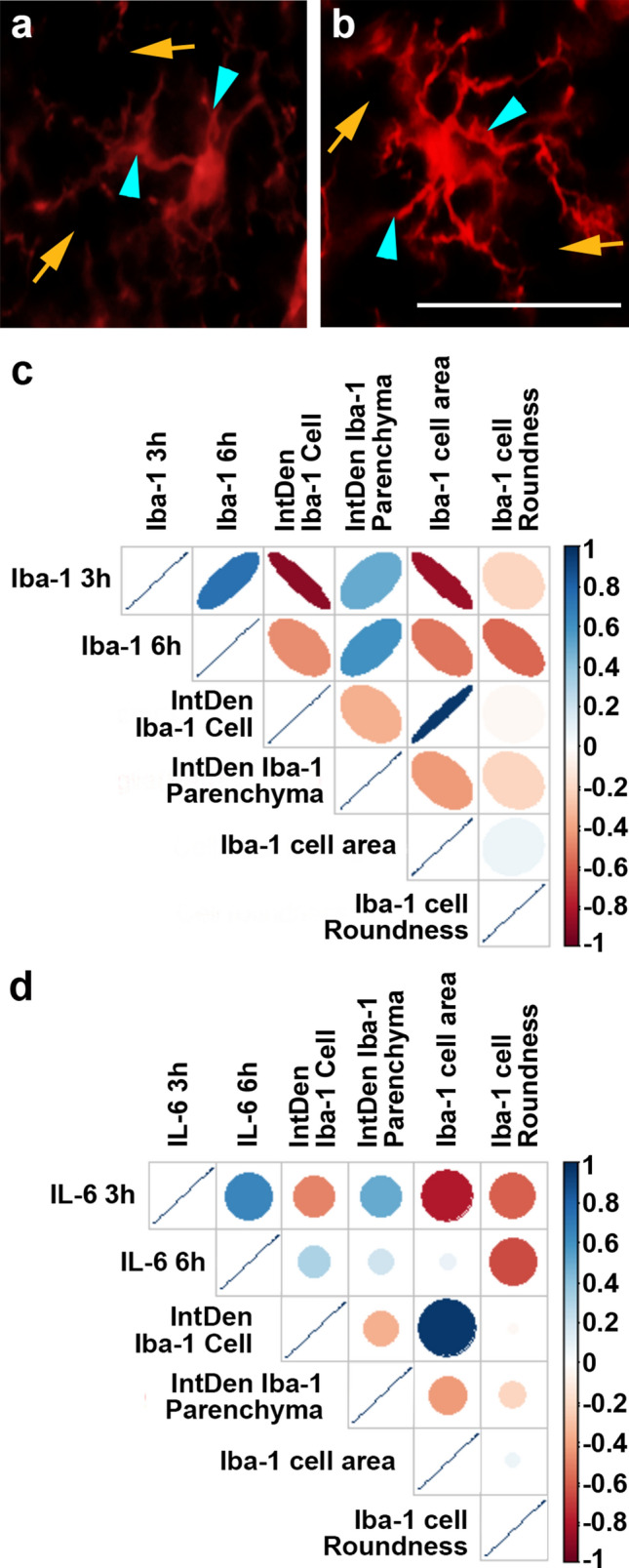
Thalamic Iba-1 + microglial/macrophage cellular IntDen/intensity and area assessed at 6 h are negatively correlated to serum levels of Iba-1 at 3 h post-injury. (**a**) Representative images demonstrating the Iba-1 + microglial cell (blue arrow heads) and the parenchyma (yellow arrows) assessed. (**c**,** d**) Correlation matrixes (generated in R^78^) depicting the Spearman Rho correlation for (**c**) Iba-1 and (**d**) IL-6 serum levels. Each pair of assessment values on the y and x axis is colored with blue indicating a stronger positive correlation, red indicating a stronger negative correlation and white being no correlation. The diameter and slant of the ovals in (**c**) indicate the significance and nature of the correlation respectively. Note that both microglial cell IntDen/intensity and cell area negatively correlate with serum Iba-1 levels at 3 h, but not 6 h post-injury. No correlations were found between serum IL-6 levels at 3 h or 6 h and any microglial/macrophage histological assessment. Scale: 20 μm.

We previously demonstrated significant microglia/macrophage activation acutely following cFPI in the thalamus of injured micro pigs that was correlated to the degree of diffuse thalamic axonal injury^29,30^. Therefore, we assessed the serum levels of microglial/macrophage-associated proteins, Iba-1 and IL-6 and correlated serum levels of each inflammatory marker with tissue immunolabeling of Iba-1 + microglial/macrophage (sham n = 3, cFPI n = 7) (Fig. 7c). Circulating levels of Iba-1 at 3 h post-injury negatively correlated with cellular IntDen/intensity (n = 10 animals; r = -0.89, p = 0.007) and Iba-1 + microglial cell area (n = 10 animals; r = -0.85, p = 0.014) but no other correlations were found with either serum levels of Iba-1.

No correlations were found between Iba-1 microglial histological assessments and IL-6 serum levels at any time post-injury (Fig. 7d). As astrocytes also secrete IL-6^41,42^, we also explored the relationship between histological features of GFAP + immunolabeling and circulating levels of IL-6, but no significant correlations were found.

### Changes in parenchymal GFAP indicate subtle thalamic astrocyte pathophysiology and correlate with circulating GFAP levels

**Figure 8:**
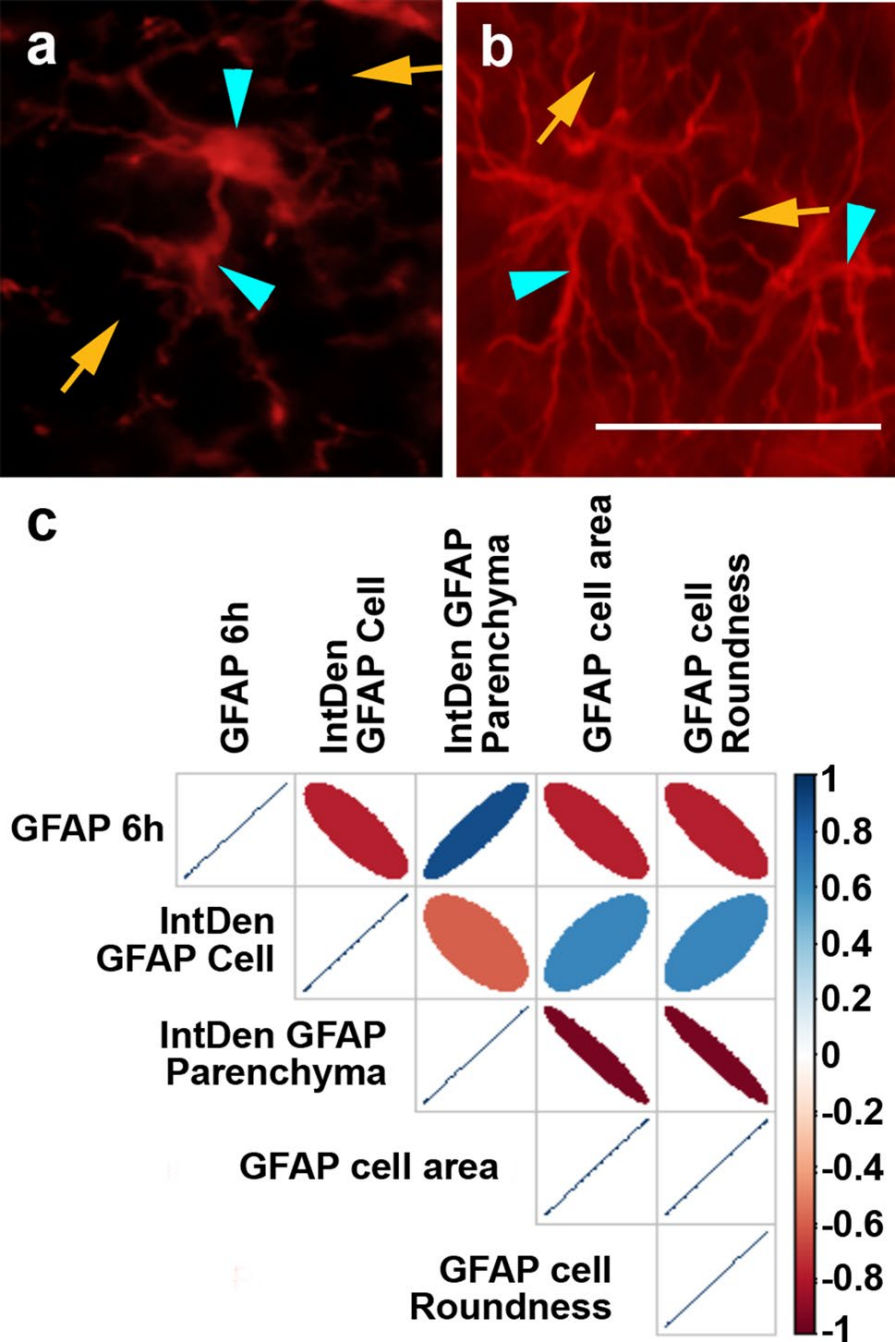
Histochemical labeling features of thalamic GFAP + astrocytes correlate with serum GFAP levels 6 h following cFPI. (**a**,**b**) Representative images demonstrating the GFAP + cell (blue arrow heads) and the parenchyma (yellow arrows) assessed. (**c**) Correlation matrix (generated in R^78^) depicting the Spearman Rho correlation for each pair of assessment values for GFAP histological and serum level readouts on the y and x axis with blue indicating a stronger positive correlation and red indicating a stronger negative correlation and white being no correlation. The diameter and slant of the ovals indicate the significance and nature of the correlation respectively. Note that GFAP serum levels at 6 h positively correlate with GFAP + parenchymal Int/Den/intensity. GFAP intensity in the parenchyma also negatively correlated with both GFAP + astrocyte cell area and roundness. Scale: 20 μm.

While, the utility of GFAP as a biomarker of gross pathological change is clearly established^16,17^, the relationship between serum GFAP levels and more subtle diffuse histopathology, especially within the thalamus, remains promising, but limited^21–24^. The cFPI model does not produce contusion in our hands^29,43^ and therefore also does not produce a gliotic scar in which astrogliosis is apparent and homogenously localized at the time point assessed. Therefore, diffuse histological characteristics of thalamic GFAP were assessed 6 h following sham or cFPI (Fig. 8). We found no significant morphological alterations of GFAP + astrocytes indicative of astrocytosis/astrocyte activation in the thalamus at 6 h following cFPI in our micro pig model (One-way-ANOVA, Bonferroni post-hoc, sham (n = 2) vs. cFPI (n = 7) animals; overall image GFAP intensity/IntDen F_1,7_ = 0.255, p = 0.629; GFAP + cell number/field F_1,7_ = 0.208, p = 0.662; astrocyte cell area F_1,7_ = 0.056, p = 0.819; astrocyte cell roundness F_1,7_ = 0.486, p = 0.508; astrocyte IntDen/intensity F_1,7_ = 0.059, p = 0.815; parenchymal IntDent/intensity F_1,7_ = 0.307, p = 0.597). To investigate potential associations between serum GFAP levels and more subtle histological features of GFAP, masks were made for both GFAP + astrocytes and parenchyma (Supplemental Fig. S1) and the histological features of GFAP were assessed in 8 micro pigs at 6 h. The IntDen/intensity of GFAP + astrocytes and thalamic parenchyma as well as the area and roundness of GFAP + astrocytes were assessed and correlated to serum GFAP levels at 6 h post-cFPI. GFAP labeling in the parenchyma varied among post-injury micro pigs as indicated in Fig. 8. Serum levels of GFAP at 6 h post-injury strongly correlated to the IntDen of the GFAP + parenchyma (n = 8 animals; r = 0.89, p = 0.019; Fig. 8c). On the other hand, the IntDen of the parenchyma negatively correlated to GFAP + cell area and roundness of GFAP + astrocytes, both indicators of cell health (GFAP + cell area n = 8 animals; r = -0.94, p = 0.005; GFAP + cell roundness r = -0.94, p = 0.005; Fig. 8). No other correlations were found.

**Figure 9:**
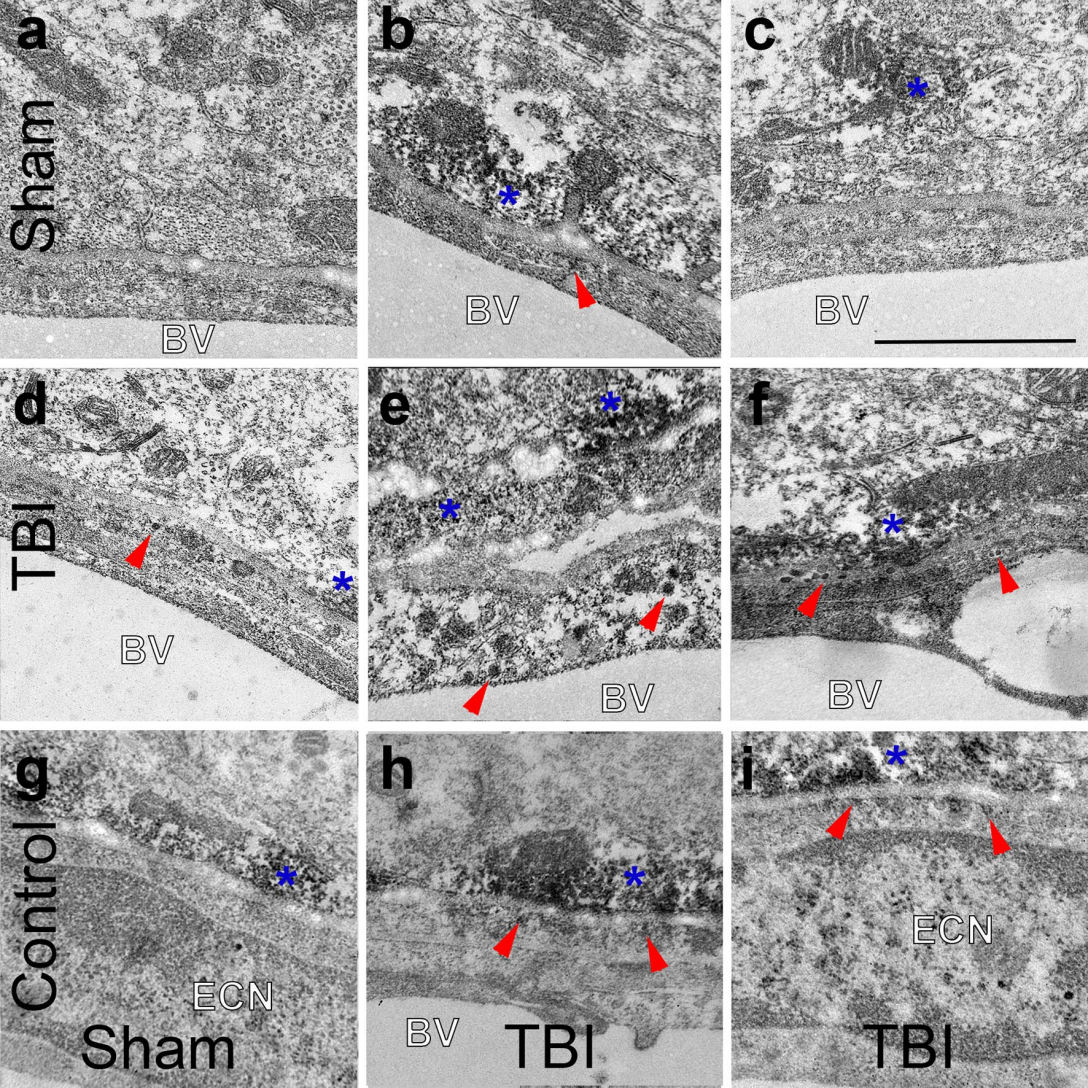
Possible transport of GFAP directly through vascular walls into the blood following TBI. Representative electron micrographs of the wall of blood vessels (BV) from the thalamus of (**a**–**c**) sham or (**d**–**f**) cFPI micro pigs labeled with GFAP (black granules). (**g**–**i**) Representative control electron micrographs in which the non-GFAP-labeled electron dense structures are not stained. While GFAP + astrocytic end feet (blue *) were seen engaged with the blood vessel in sham-injured micro pigs, this engagement was more pronounced following cFPI. GFAP-containing vesicles (red arrow heads) were also observed in the basement membrane and in the endothelial cell cytoplasm indicating a possible mode of GFAP transport from the brain into the blood through the walls of the vasculature. ECN = endothelial cell nucleus. Scale: 2 μm.

To further investigate the correlation between parenchymal GFAP IntDen/intensity and post-injury serum GFAP levels, ultrastructural assessment of GFAP localization within the thalamus was performed. Owing to the BBB disruption observed in our model and considering that astrocytic end feet are tightly associated with the vasculature, forming part of the BBB^44^, we further focused our investigation on the walls of thalamic blood vessels. While areas of the vasculature were devoid of astrocyte association in both sham and injured thalami, GFAP + astrocytic end feet were observed associated with most vessels in sham-injured micro pigs (Fig. 9a–c ), however, GFAP + astrocyte end feet appeared more apparent in thalamic vessels following cFPI (Fig. 9d–f ). Importantly, vesicles containing GFAP were identified in the blood vessel walls of thalamic vasculature, both within the cytoplasm of endothelial cells (Fig. 9d,e ) as well as the basement membrane of the blood vessel wall (Fig. 9f ) indicating a potential endocytosis of GFAP by endothelial cells for transport to the blood. No GFAP + vesicles were observed within the walls of the thalamic vasculature following sham-injury.

## Discussion

Serum biomarkers have been touted as promising non-invasive tools to enhance diagnostic, prognostic, and pharmacodynamic responses (a.k.a., theranostics) information following brain injury. Neuronal UCH-L1 and astrocytic GFAP are relatively well-established biomarkers of TBI. Many studies have demonstrated the association of UCH-L1 and GFAP levels in serum with moderate or severe TBI^34,36^. Levels of GFAP within the serum have been linked to ensuing gross brain pathology, such as lesions, contusions and hemorrhages^45^, however, as our cFPI model of mTBI in micro pigs does not produce such focal injuries^29^, the change in serum GFAP following cFPI cannot be associated with gross pathologies. Clinical studies have demonstrated that the levels of circulating GFAP within the first day post-injury could also be associated with the presence of more subtle pathologies, including DAI^21–23^. Experimental blast-wave-induced TBI has also shown associations with elevated GFAP serum levels^46^, however, a clinical study found a negative correlation between serum GFAP levels and blast TBI^47^, highlighting the complexity of serum biomarker investigations. Direct correlations between serum biomarker levels and the specific alterations of diffuse histopathologies, especially within the thalamus, remain understudied.

In the present study, we used a well-established model of cFPI in adult micro pigs to characterize specific neuropathological and biomarker changes 6 h following mTBI. We found that GFAP levels increased by eightfold in the serum within the initial 6 h following TBI as compared to pre-injury or immediately post-injury. These data nicely recapitulate the changes in GFAP serum levels observed following TBI in patients^17,34,48^. The temporal profile of GFAP serum levels in the micro pig is also consistent with our previously published serum GFAP levels in rodent models of TBI^8^. Although we explored the relationship between serum biomarkers and thalamic injury, we recognize that in a model of diffuse injury like cFPI, the increases in GFAP that were observed almost certainly reflect a more global process. There was, however, no change in serum UCH-L1, Iba-1 or IL-6 levels following TBI compared to baseline. Although we specifically explored the relationship between serum biomarkers and diffuse thalamic injury, we recognize that the increases in GFAP that were observed almost certainly reflect a more global process and will require assessments of other brain regions.

As published previously, while significant microscopic changes, including glial activation and DAI were evident in the thalamic domain, no overt tissue damage or gross pathology were apparent^29^. In the current study we also found accumulation of GFAP in the parenchyma and in vesicles within the vasculature walls, in the thalamus. Importantly, our study demonstrates that morphological changes that are potentially indicative of abnormal/injured astrocytes correlate strongly with the degree of thalamic GFAP parenchymal accumulation, which, in turn, is marked by increased circulating GFAP levels (Fig. 8). Such dynamics suggest that increased serum GFAP concentrations acutely after diffuse mTBI could indicate potential diffuse thalamic injury. In particular, it seems plausible that the pathological interstitial accumulation of GFAP proteins in the thalamus presumably occurs as a result of cellular GFAP leakage from wounded astrocytes, as previously described by the Wanner lab^49^. This study suggested that the release of GFAP could be an indicator/predecessor of future astrocyte cell death^49^. A seminal study by Di Pietro et al.^50^ reported similar observations that GFAP release preceded subsequent cell death in an in vitro model of graded stretch injury induced in rat organotypic hippocampal slice cultures^50^. However, the studies from the Wanner lab also found that a subset of wounded human astrocytes demonstrated membrane leakiness without overt morphological alterations following an in vitro stretch injury whereas significant morphological alterations paired with GFAP release signified ultimate cell death^49^. Based on our previous findings that there are no indications of cell death in the thalamus of cFPI pigs^29^, paired with our current finding that astrocyte morphology is not significantly altered in the thalamus following cFPI (Fig. 8), it is unlikely that we are observing astrocytes immediately prior to death. Our findings also raise the potential that GFAP is being directly transduced through the walls of the vasculature into the blood (Fig. 9). Taken together, these findings suggest that circulating GFAP can be a powerful tool to identify subtle injury after diffuse mTBI and also that better understanding of GFAP kinetics might eventually provide strategies to characterize optimal therapeutic time windows for changing the fate of injured astrocytes after diffuse mTBI.

Interestingly, circulating GFAP strongly correlated with GFAP parenchymal accumulation in a relatively restricted area of the brain, namely the thalamus. It is likely that the processes observed in the thalamus after mTBI produced by cFPI reflects a broader brain condition. On the other hand, the relation between thalamic extracellular GFAP and circulating GFAP after mTBI is clearly complex. Serum biomarker levels are likely a function of the type of mTBI and possibly involve several distinct clearance pathways such as the glymphatic system. To this end, our data demonstrate that BBB disruption can also occur acutely following cFPI in the micro pig, in which there is no pericontusional/focal lesion. This is in alignment with experimental and human studies showing BBB disruption following mTBI^39^. This BBB breakdown in the absence of an expanding lesion and overt tissue damage could reflect minor astrocyte alterations that do not signify astrogliosis. The BBB is, in part, maintained by the perivascular sheath, which is formed by both astrocytic end feet and enveloping pericytes^51^. Previous studies have shown that GFAP + astrocytic end feet increase association with the vasculature following TBI^52–54^. We also noted a potential increase in the appearance of GFAP + end feet around thalamic blood vessels in our cFPI model of mTBI. Importantly, we found a significant correlation between the IntDen/intensity of GFAP in the parenchyma and GFAP serum biomarker levels, indicating potential release or leakage of GFAP from astrocytes into the parenchyma following mTBI (Fig. 8). We also found preliminary evidence of GFAP + vesicles within the walls of the blood vessels, indicating endocytosis of parenchymal GFAP by endothelial cells (Fig. 9). Endothelial cells have been shown to transport albumin and other macro-molecules via calveolin-mediated endocytosis^55,56^. Therefore, based on our findings, it appears that GFAP may be transported directly from the brain into the blood through the endothelial cells lining the blood vessel walls via vesicular transport following cFPI. This, however, remains speculative and this possibility represents a critical area that requires further study.

Based on previous clinical assessments indicating that UCH-L1 levels in serum could be linked to diffuse pathologies following TBI^17,20^, it was surprising that UCH-L1 levels did not change in our micro pig model of mTBI. This could be due to the nature of higher order animal work, which involves a higher level of complexity with resultant increased animal to animal variability. This higher variability reduces the likelihood of observing significant changes following injury with the small sample sizes generally used in large animal studies, including the current work, and contrasts the clinical studies of serum biomarkers after mTBI—which are carried out in large samples^10^. It is also possible that the level of injury used in our studies is less than that observed in the patients in clinical studies and/or that cFPI produces an injury pattern that highlights axonal and/or glial rather than neuronal injury. The lack of any notable trend toward increased UCH-L1 levels following injury, however, suggests that it is unlikely that inter-animal variability is the primary reason for our negative findings (Fig. 2). It is also possible that the biomarker assay performance for UCH-L1 is less optimal in micro pigs than for humans. Although there is a 98% homology between human and pig UCH-L1 amino acid sequences^57^, the large number of samples below the detectable limit in this study suggests that lack of specificity for pig UCH-L1 is a possibility. Finally, there are also clinical findings that demonstrated no utility for UCH-L1 serum biomarker levels following TBI. Studies from the ProTECT-III trail found that while incorporating GFAP serum levels significantly predicted negative outcomes 6 months following TBI, incorporating UCH-L1 levels did not improve prognostic ability^36^. Studies have also shown that GFAP and UCH-L1 serum levels do not correlate in the clinical population^34^. Our lackluster UCH-L1 findings are in alignment with these negative clinical studies.

Serum levels of Iba-1 and IL-6 were also assessed to explore their potential as serum biomarkers of neuroinflammation, a common pathology following TBI^58–62^, and a pathology that is present in the thalamic domain of cFPI micro pigs^29^. We found that serum levels of Iba-1 at 3 h were negatively correlated to both Iba-1 + microglia/macrophage cellular intensity/IntDen and Iba-1 + cell area (Fig. 7). Neither of these correlations, however, were observed when comparing serum Iba-1 levels at 6 h to Iba-1 + microglia/macrophage histological features. These changes in Iba-1 + microglia are indicative of the morphological features of activated microglia/macrophage that were assessed in our previous study^29^. Non-reactive, surveying microglia/macrophage cover large areas with highly ramified process networks^63–65^. Upon activation microglia/macrophage processes shorten and thicken substantially, drastically reducing the area that the cell covers^66,67^. Studies have also shown that expression of Iba-1 increases in activated microglia/macrophage as compared to their non-reactive surveying counterparts^68^. This is a likely possibility, as our previous studies demonstrated notable microglia/macrophage activation in the thalamus 6 h following cFPI^30^. The correlations between histology and serum levels at 3 h but not at 6 h, could also indicate a potential predictive element to Iba-1 serum levels in terms of future microglia/macrophage activation, however, this possibility remains to be fully investigated.

There was also a significant correlation between microglia/macrophage cellular IntDen and the area of the microglia/macrophage cells (Fig. 7). This correlation could be due to the fact that cellular area is used to calculate IntDen and therefore these two metrics are inextricably linked. However, there was no observable correlation between GFAP + cellular IntDen and astrocyte cell area (Fig. 8), indicating that the correlation between Iba-1 + cell IntDen and Iba-1 + cell area may not merely be a function of the formula used to calculate IntDen. As both increased cellular expression of Iba-1, which is reflected in the cellular IntDen, and decrease in microglial/macrophage cellular area are characteristics of activated microglial/macrophage , it is likely that the association between these two metrics is indicative of microglial/macrophage activation. This correlation between Iba-1 + microglial cell intensity/IntDen, cell area and serum Iba-1 levels could potentially be leveraged to develop higher throughput assessments of microglial/macrophage activation following TBI, however, it would require further study into the correlations between these metrics and the common morphological alterations occurring in activated microglial/macrophage in a more systematic fashion.

Serum levels of the cytokine, IL-6, did not correlate with any histological feature related to Iba-1 + microglial/macrophage or GFAP + astrocytes. Histological features for both microglial/macrophage and astrocytes were correlated to IL-6 serum levels, as both microglial/macrophage and astrocytes have been shown to secret IL-6^41,69–71^. This was surprising, as IL-6 has previously been linked to BBB disruption, which is one of the likely routes of protein movement out of the brain parenchyma into the blood following TBI. Addition of IL-6 to endothelial cells in culture drastically reduced the trans endothelial electrical resistance, which would negatively affect BBB integrity^72^. Additionally, constitutive expression of IL-6 in transgenic mice resulted in impaired BBB formation^41^. It is possible that serum levels of IL-6 could be an effective indication of BBB breakdown in a more severe TBI, in which gross tissue damage results in much more substantial BBB disruption. Due to the transient nature of BBB permeability in mTBI produced by cFPI, it is also possible that we missed this potential correlation between IL-6 and BBB opening due to the timing of the current study; longitudinal assessments would be needed to fully evaluate this possibility. Once again, our limited sample, the injury level chosen, and variability might be limiting our ability to detect associations. However, as is the case with experimental studies of thalamic damage following TBI^30,73–75^, longitudinal assessments will be needed to more fully evaluate the pathophysiological mechanisms of mTBI and their potential contributions to chronic thalamic dysfunction.

In the present study, we demonstrate the potential use of circulating biomarkers to assess microscopic histopathological progressions in the thalamus following mTBI.

Considering the strategic role of the thalamus in brain function and consistent involvement following brain injury, our findings suggest that in diffuse injury, monitoring serum GFAP and Iba-1 might provide clinically relevant insights into the underlying pathophysiology and biomarker release kinetics following mTBI, providing previously underappreciated diagnostic capability. Future studies are, however, needed to determine if serum GFAP and/or Iba-1 could provide insight into the impact of therapies on thalamic injury and/or more global diffuse brain pathology after mTBI.

## Methods

### Animals

We used a total of seventeen adult male Yukatan micro pigs (n = 3 sham; n = 14 injured), weighing 15–25 kg (~ 6 months of age). Other data generated from these animals have been previously reported^29^. Animals were housed in environmentally controlled pens in pairs on a 12 h light–dark cycle, with free access to food and water. All experimental protocols were approved by the Virginia Commonwealth University Institutional Animal Care and Use Committee, an AAALAC international accredited organization. Experiments were conducted in accordance with the Virginia Commonwealth University institutional guidelines concerning the care and use of laboratory animals (Institutional Animal Care and Use Committee), which adhere to regulations including, but not limited to, those set forth in the “Guide for the Care and Use of Laboratory Animals: 8^th^ Edition” (National Research Council).

### Surgical preparation and injury induction

As previously published^29^, micro pigs were initially anesthetized with an intramuscular injection of 100 mg/ml Xylazine (2.2 mg/kg; AnaSed Injection, Shenondoah, IA, USA) and 100 mg/ml Telazol (2.0 mg/kg; Tiletamine HCL and Zolazepam HCL; Pfizer, New York, NY, USA) followed by intravenous administration of sodium pentobarbital (60 mg/kg; Sigma-Aldrich, St. Louis, MO, USA). Once the absence of a corneal reflex was verified, the micro pig was intubated and ventilated with 1–2% isoflurane mixed in 100% oxygen throughout the experiment. Ophthalmic lubricant (Dechra, Overland Park, KS, USA) was applied to avoid damage or drying of the eye. Body temperature was monitored with a rectal thermometer and maintained at 37 °C with a heating pad. Catheters were placed in the right femoral artery and vein for continuous monitoring of mean arterial blood pressure (MABP), assessment of blood gases, and infusion of drug treatment, as described below, or Lactated Ringer’s (Hospira, Lake Forest, IL, USA) to maintain hydration. A midline incision was made from the supraorbital process to the nuchal crest and a 14 mm diameter circular craniotomy was trephined along the sagittal suture, positioning the center of the craniotomy 15 mm anterior to lambda (on the nuchal crest) and leaving the Dura intact. A stainless steel custom threaded hub (Custom Design and Fabrication, Richmond, VA, USA) was screwed into the craniotomy site to a depth of ~ 4 mm. Screws were then placed directly posterior and anterior-lateral to the craniotomy and dental acrylic (methyl-methacrylate; Hygenic Corp., Akron, OH) was applied around the hub and screws to insure hub stability. The induction of the cFPI was done as described previously^29,30^. Briefly, anesthetized micro pigs were connected to a cFPI device retrofitted with an L-shaped stainless-steel adaptor that allowed for a sealed connection to the injury hub. Micro pigs were then injured at a magnitude of 1.7 ± 0.2 atmospheres with a pressure pulse measured by a transducer affixed to the injury device and displayed on an oscilloscope (Tektronix, Beaverton, OR, USA). Immediately after injury induction, animals were disconnected from the injury device, the screws and hub were removed from the bone and the dental acrylic, hub and screws were removed en bloc. This injury did not result in any breach of the Dura mater. Gel foam was placed over the craniotomy/injury site, to alleviate small amounts of bone bleeding, and the scalp was sutured. Animals were anesthetized for the duration of the 6 h post-injury monitoring period.

Systemic physiology was assessed prior to injury and throughout the 6 h post-injury monitoring period. Heart rate, arterial blood pressure, rectal temperature, and hemoglobin oxygen saturation were monitored and recorded throughout the experiment via a Cardell MAX-12HD (Sharn Veterinary, Inc., Chicago, IL, USA). The partial pressures of oxygen and carbon dioxide in arterial blood, PaO_2_ and PaCO_2_, respectively, and pH values were assessed using a Stat Profile pHOx (NOVA Biomedical, Waltham, MA, USA). The resting PaCO_2_ level was maintained between 35-40 mmHg by adjusting the rate and/or tidal volume of the respirator. All animals maintained physiological homeostasis (i.e. 60 mmHg < MABP < 130 mmHg, hemoglobin oxygen saturation > 90%, 90BPM < Heart rate < 140 BPM). Physiological results for these animals were published previously^29^.

### Detection and quantification of serum biomarker levels

Serial arterial blood samples were obtained pre-cFPI, as well as at 1 min, 30 min, 1 h, 3 h and 6 h post-injury. Blood volume was replaced with intravenous infusion of Lactated Ringer’s (Hospira, Lake Forest, IL, USA) to maintain euvolemia. All blood samples were processed to obtain serum according to the OBTT manual of standard operating procedures (MSOP^76^). Briefly, 3 ml of arterial blood was collected in a vacutainer blood collection tube (Cat#367,981; Becton Dickinson, Franklin Lakes, NJ, USA), inverted 5 times followed by a 30 min incubation at room temperature. The serum was separated by centrifugation at 3,900 RPM for 15 min on an EBA20 clinical centrifuge (Hettich; Tuttligen, Germany) and stored at -80 °C. Successively, blind samples were shipped to Banyan Biomarkers and University of Florida for analysis.

Serum levels of GFAP and UCH-L1 were measured by enzyme-linked immunosorbent assay (ELISA) using proprietary anti-GFAP and anti-UCH-L1 antibodies at Banyan Biomarkers, as previously described^8,37^. Please see Mondello and associates and Shear and colleagues for a more detailed description of the ELISA and biomarker-related methods used by Banyan Biomarkers in these studies^8,37^. Serum levels of Iba-1 and IL-6 were measured at the University of Florida using Porcine AIF1 (Allograft Inflammatory Factor 1)/Iba-1 ELISA Kit (Iwai-chem; # E-EL-P2172) and Human IL-6 Quantikine ELISA Kit (R&D System, # D6050), respectively according to manufacturer’s instructions. All animal’s serum was assessed for GFAP and UCH-L1 over time (n = 3 sham and n = 14 cFPI), however, as sample quantity was limited, some samples were not available for assessment of Iba-1 (n = 3 sham and n = 12 cFPI) and IL6 (n = 3 sham and n = 11 cFPI).

### Tissue processing

At 6 h after cFPI micro pigs were overdosed with 3 ml euthasol euthanasia-III solution (Henry Schein, Dublin, OH; USA) transcardially perfused with 0.9% saline followed by 4% paraformaldehyde/0.2% gluteraldehyde in Millonig’s buffer (136 mM sodium phosphate monobasic/109 mM sodium hydroxide) for immunohistochemical analysis, as previously published^29^. After transcardial perfusion, the brains were removed and post-fixed in 4% paraformaldehyde/0.2% gluteraldehyde/Millonig’s buffer for 36-48 h. Postfixed brains were blocked into 5 mm coronal segments throughout the rostral-caudal extent using a tissue slicer (Zivic Instruments, Pittsburgh, PA, USA). Segments containing the thalamus were bisected at the midline and the left side was analyzed. The 5 mm coronal segments containing the thalamus were coronally sectioned in 0.1 M phosphate buffer with a vibratome (Leica, Banockburn, IL, USA) at a thickness of 40 µm. Sections were collected serially in 6-well plates (240 μm between sections in each well) and stored in Millonig's buffer at 4 °C. For immunohistological quantification, a random well (1–6) was selected using a random number generator and four sections, representing the rostral-caudal axis contained within the selected well, were analyzed. All histological analyses were restricted to the thalamus using anatomical landmarks and were performed by an investigator blinded to animal identity.

### Immunohistochemistry

To visualize serum albumin within the brain parenchyma, indicative of blood brain barrier (BBB) disruption, sections were blocked and permeabilized in 1.5% triton/5% NGS/PBS followed by overnight incubation with a primary antibody against pig serum albumin (1:1,000; Cat.#ab79960, abcam, Cambridge, MA, USA) in 5% NGS/PBS at 4C°. A biotinylated goat anti-rabbit IgG (1:1,000; Cat.# BA-1000, Vector Laboratories, Burlingame, CA, USA) secondary antibody was used. The sections were then incubated in avidin biotinylated enzyme complex using the Vectastain ABC kit (Vector Laboratories, Burlingame, CA, USA) followed by visualization with 0.05% diaminobenzidine/ 0.01% H_2_O_2_/0.3% imidazole/PBS. The tissue was mounted, dehydrated, and cover-slipped. Visualization of serum albumin was performed using a Nikon Eclipse 800 microscope (Nikon, Tokyo, Japan) equipped with an Olympus DP71 camera (Olympus, Center Valley, PA, USA).

For fluorescent immunohistochemistry, archival tissue that had previously been immuno-labeled for each marker was used. All tissue was immuno-labeled in the same run to reduce variability, however, this archival tissue did not include all animals assessed for serum biomarker levels and therefore the ns for histological correlations are variable. Sections containing micro pig thalamus were labeled with the following antibodies: rabbit anti-C-terminus of β-APP (1:700; Cat.# 51–2,700, Life Technologies, Carlsbad, CA, USA), rabbit anti-UCH-L1 (1:2,000; Cat.# 11,896, Cell Signaling Technology, Danvers, MA, USA), rabbit anti-Iba-1 (1:1,000; Cat.# 019–19,741, Wako, Osaka, Japan), and mouse anti-GFAP (1:1,000; Cat.#MAB3402, Millipore Burlington, MA, USA). The tissue was blocked with 5% NGS/2% BSA/1.5% Triton/PBS followed by incubation with the anti-UCH-L1, anti-Iba-1 or anti-GFAP antibody overnight at 4 °C. Alexa Fluor 568-conjugated goat anti-rabbit IgG (1:700; Cat.# A-11011, Life Technologies, Carlsbad, CA, USA) or Alexa Fluor 568-conjugated goat anti-mouse IgG (1:700; Cat.# A-11004, Life Technologies, Carlsbad, CA, USA) was used to visualize the immunolabeling. Labeled tissue was mounted using Vectashield hardset mounting medium with DAPI (Cat.# H-1500; Vector Laboratories, Burlingame, CA, USA). Tissue sections were processed concomitantly and microscope settings were held consistent to obviate variability in intensity based on staining or imaging differences. Immunolabeling for all tissue was done at the same time for each individual marker to reduce run-to-run variability. The animals included for each histological assessment differed in some cases owing to the availability of tissue at the time of labeling. Visualization was performed using a Nikon Eclipse 800 microscope (Nikon, Tokyo, Japan) equipped with an Olympus DP71 camera (Olympus, Center Valley, PA, USA).

### Quantitative assessment of histological alterations

Images were taken by an investigator blinded to animal identification and group at 20X magnification in a systematically-random fashion using Dapi to verify focus and restriction within the thalamic region of interest. Images were duplicated and the duplicates were processed with background subtraction and automatic thresholding to generate masks of UCH-L1 + neurons, Iba-1 + microglia and GFAP + astrocytes (Supplemental Fig. S1). All cells within the mask were added to the Region of Interest (ROI) Manager in FIJI. Measurements of the number, area, average fluorescence intensity/integrated density (IntDen) and solidity/complexity of individual cells were assessed using the Measure function on the original micrograph without any image processing. Successively, the cellular mask was inverted to generate a mask of the non-cellular parenchyma (Supplemental Fig. S1). The IntDen of the parenchyma was then assessed in a similar fashion as the cellular IntDen using the ROI Manager and Measurement functions in FIJI on the original non-processed image. Localization of UCH-L1 within either the nucleus or the cytoplasm of individual neurons was assessed in 30 randomly selected neurons per animal (5 neurons/image; 1 image/section) by eye.

Assessment of the degree of DAI in the thalamic domain of animals used for the current study was published previously^29^. To assess axonal injury using APP labeling, fluorescent intensity threshold was set for all images to eliminate any neuronal somatic expression of APP from the assessment. The number of APP^+^ axonal swellings was analyzed using the particle analysis function in ImageJ software (NIH, Bethesda, MD). The number of APP^+^ swellings per unit area was quantified for each image and averaged for each animal.

Additionally, the level of microglial activation in the thalamus of animals used for this current study were also published previously^29^. For this analysis a microglial activation index was assigned to Iba-1-labled tissue. Briefly, the degree of microglia activation was assessed using a graded scale from 0–5 (0 = no microglial activation observed, 1 = ramified microglia with thicker processes and darker Iba-1 labeling observed in ~ 5% of the thalamus, 2 = activated microglia observed in ~ 5–10% of the thalamus, 3 = activated microglia observed in ~ 10 < 25% of the thalamus, 4 = activated microglia observed in ~ 25 < 50% of the thalamus and 5 = activated microglia observed in > 50% of the thalamus).

### Electron microscopy

To investigate the localization of GFAP in relation to the vascular walls of thalamic blood vessels 6 h following cFPI, immune-electron-microscopy was performed as done previously^29^. Briefly, a subset of tissue was immunolabeled with mouse anti-GFAP (1:1,000; Cat.#MAB3402, Millipore Burlington, MA, USA) followed by incubation with biotinylated goat anti-mouse IgG (1:1,000; Cat# BA-9200, Vector Laboratories, Burlingame, CA, USA) secondary antibody. The reaction product was visualized with 0.05% diaminobenzidine/0.01% hydrogen peroxide/0.3% imidazole in 0.1 M phosphate buffer and the tissue was prepared for EM analysis. In this approach, tissue sections were osmicated, dehydrated, and embedded in epoxy resin on plastic slides. After resin curing, the slides were studied with routine light microscopy to identify thalamic areas demonstrating GFAP labeling for excision. These regions of interest were selected randomly for each animal. These sites were removed, mounted on plastic studs, and 40-70 nm sections were cut serially and mounted on Formvar-coated slotted grids. The grids were stained in 5% uranyl acetate in 50% methanol and 0.5% lead citrate. One grid for each ROI was left unstained to reduce the resolution of non-immunolabeled cellular ultrastructure as a control. These control grids were not stained with 5% uranyl acetate in 50% methanol and 0.5% lead citrate thus reducing the contrast for non-DAB-labeled cellular structures. Ultrastructural qualitative analysis was performed using a JEOL JEM 1,230 transmission electron microscope (JEOL-USA, Peabody, MA, USA) equipped with Ultrascan 4000SP CCD and Orius SC1000 CCD cameras (Gatan, Pleasanton, CA, USA). All blood vessels within one grid for each animal were visualized for localization of GFAP, however, only those that were clear of dirt or holes in the membrane were imaged for further assessment. Images were taken at 1000kv with auto-focus and auto-exposure at various magnifications.

### Statistical analysis

Exploratory analysis was carried out to determine the distribution of the data.

For unpaired group comparison the Mann– Whitney U-test was used. For comparisons across time points the Friedman test was used. For comparisons between sham and cFPI a One-way ANOVA with Bonferroni post hoc correction for multiple comparisons was used. A Spearman’s Rho correlation analysis was conducted for each histological assessment and each serum biomarker sample throughout the experiment. Associations were considered significant if the Benjamini Hochberg (FDR)^77^ adjusted p-values were less than 0.05. All hypothesis tests conducted were two-tailed, and a p value < 0.05 was considered significant. Statistical analysis was performed using SAS (SAS version 9.4, SAS Institute Inc., Cary, NC, USA and R^78^ (https://www.r-project.org, version 3.5.1) in RStudio (https://www.rstudio.com, version 1.1.456).

## Supplementary information

Supplementary Figure S1.

## Data Availability

All data presented herein, and protocols used to generate the data will be made available to qualified persons upon reasonable request.
