# Supplementary material for: Circulating GFAP and Iba-1 levels are associated with pathophysiological sequelae in the thalamus in a pig model of mild TBI
Source: Sci Rep. 2020 Aug 7;10:13369. doi: 10.1038/s41598-020-70266-w (PMC7415146; doi:10.1038/s41598-020-70266-w)
Supplement: Supplementary file 1 — Supplementary Figure S1. [file 41598_2020_70266_MOESM1_ESM.pdf]

Circulating GFAP and Iba-1 levels are associated with pathophysiological sequelae of mild TBI in a pig model: an OBTT study

Audrey D. Lafrenaye[1*], Stefania Mondello[2,3*], Kevin K. Wang[4], Zhihui Yang[4], John T. Povlishock[1], Karen Gorse[1], Susan Walker[1], Ronald L. Hayes[5], Patrick M. Kochanek[6]

[1] Department of Anatomy and Neurobiology, Virginia Commonwealth University, Richmond, VA

[2] Department of Biomedical and Dental Sciences and Morphofunctional Imaging, University of Messina, Messina, Italy

[3] Oasi Research Institute-IRCCS, Troina, Italy

[4] Center for Neuroproteomics & Biomarkers Research, Departments of Psychiatry & Neuroscience, University of Florida, Gainesville, FL

[5] Banyan Biomarkers, Inc., Alachua, FL

[6] Department of Critical Care Medicine, University of Pittsburgh, Pittsburgh, PA

* Co-first authors

Corresponding Author:
Audrey D. Lafrenaye, Ph.D.
Department of Anatomy and Neurobiology
Virginia Commonwealth University Medical Center
P.O. Box 980709, Richmond, Virginia 23298
Phone: 804.828.4435
Fax: 804.828.9477
Email: Audrey.Lafrenaye@vcuhealth.org

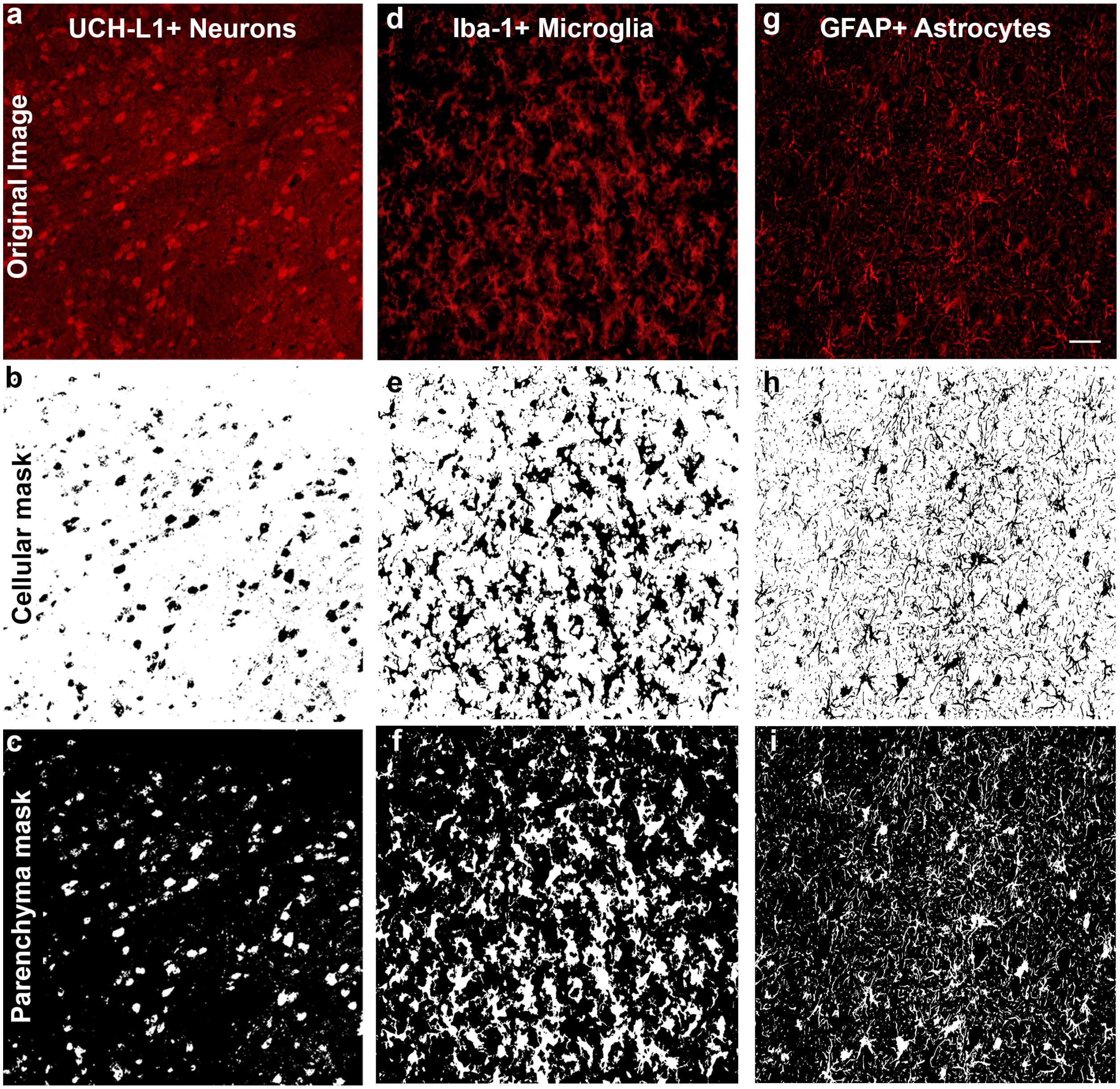

Supplemental Figure S1 Representative images depicting the workflow for histological assessment of thalamic neurons and glia in micro pigs 6h post-sham or cFPI. Histochemical labeling of thalamic **(a-c)** UCH-L1, **(d-f)** Iba-1 and **(g-i)** GFAP 6h following cFPI. The top panel **(a, d, g)** represent original epifluorescent photomicrographs of **(a)** UCH-L1, **(d)** Iba-1 and **(g)** GFAP captured under consistent imaging parameters for all animals imaged with each label. Thresholding was done to create a region of interest (ROI) mask for **(b)** UCH-L1+ neurons or **(e)** Iba-1+ or **(h)** GFAP+ glia followed by inversion of the cellular mask to create a **(c, f, i)** ROI mask for measurements of the non-cellular parenchyma for each label. Scale: 20µm.

Supplemental Figure Legends

Supplemental Figure S1 Representative images depicting the workflow for histological assessment of thalamic neurons and glia in micro pigs 6h post-sham or cFPI. Histochemical labeling of thalamic **(a-c)** UCH-L1, **(d-f)** Iba-1 and **(g-i)** GFAP 6h following cFPI. The top panel **(a, d, g)** represent original epifluorescent photomicrographs of **(a)** UCH-L1, **(d)** Iba-1 and **(g)** GFAP captured under consistent imaging parameters for all animals imaged with each label. Thresholding was done to create a region of interest (ROI) mask for **(b)** UCH-L1+ neurons or **(e)** Iba-1+ or **(h)** GFAP+ glia followed by inversion of the cellular mask to create a **(c, f, i)** ROI mask for measurements of the non-cellular parenchyma for each label. Scale: 20μm.
